# Carbon fluxes related to land use and land cover change in Baden-Württemberg

**DOI:** 10.1007/s10661-023-11141-9

**Published:** 2023-04-27

**Authors:** Veit Ulrich, Michael Schultz, Sven Lautenbach, Alexander Zipf

**Affiliations:** 1 Institute of Geography, Heidelberg University, Im Neuenheimer Feld 348, Heidelberg, 69120 Germany; 2 Institute of Geography, Tübingen University, Rümelinstr. 19-21, Tübingen, 72070 Germany; 3 Heidelberg Institute for Geoinformation Technology gGmbH, Berliner Str. 45, Heidelberg, 69120 Germany

**Keywords:** Land use change, Land cover change, Carbon fluxes, OpenStreetMap

## Abstract

Spatially explicit information on carbon fluxes related to land use and land cover change (LULCC) is of value for the implementation of local climate change mitigation strategies. However, estimates of these carbon fluxes are often aggregated to larger areas. We estimated committed gross carbon fluxes related to LULCC in Baden-Württemberg, Germany, using different emission factors. In doing so, we compared four different data sources regarding their suitability for estimating the fluxes: (a) a land cover dataset derived from OpenStreetMap (OSMlanduse); (b) OSMlanduse with removal of sliver polygons (OSMlanduse cleaned), (c) OSMlanduse enhanced with a remote sensing time series analysis (OSMlanduse+); (d) the LULCC product of Landschaftsveränderungsdienst (LaVerDi) from the German Federal Agency of Cartography and Geodesy. We produced a high range of carbon flux estimates, mostly caused by differences in the area of the LULCC detected by the different change methods. Except for the OSMlanduse change method, all LULCC methods achieved results that are comparable to other gross emission estimates. The carbon flux estimates of the most plausible change methods, OSMlanduse cleaned and OSMlanduse+, were 291,710 Mg C yr^-1^ and 93,591 Mg C yr^-1^, respectively. Uncertainties were mainly caused by incomplete spatial coverage of OSMlanduse, false positive LULCC due to changes and corrections made in OpenStreetMap during the study period, and a high number of sliver polygons in the OSMlanduse changes. Overall, the results showed that OSM can be successfully used to estimate LULCC carbon fluxes if data preprocessing is performed with the suggested methods.

## Introduction

Our planet is currently impacted by climate change, which is mostly caused by greenhouse gas (GHG) emissions linked to human activity (Friedlingstein et al., 2022; IPCC, 2022). One of the most important greenhouse gases is CO_2_ (Friedlingstein et al., 2022). Land use and land cover change (LULCC) is a significant driver of anthropogenic CO_2_ emissions (Friedlingstein et al., 2022). To reduce carbon fluxes related to LULCC and implement emission mitigation strategies on the regional scale, for instance through carbon sequestration (Friedlingstein et al., 2022; IPCC, 2022), knowledge of the occurrence of these fluxes is required at local scale. Spatially explicit estimation of carbon fluxes allows for connecting measurements of these fluxes with the underlying emission sources. Spatially explicit data can also provide information at scales relevant to various climate-related policies, programs and stakeholders. In this way, they can be beneficial for governments or non-governmental actors aiming to implement mitigation strategies such as reforestation (Harris et al., 2021).

Many studies have estimated carbon fluxes linked to LULCC (Fuchs et al., 2016; Gasser et al., 2020; Gensior et al., 2022; Harris et al., 2012; Harris et al., 2021; Houghton & Nassikas, 2017; Hutyra et al., 2011; Lam et al., 2021; Smith & Rothwell, 2013). These studies can be divided into studies specifically assessing emissions (gross emissions) and studies assessing emissions and sinks (net emissions). It is important to note this difference because this study estimates gross emissions, while the majority of studies assessing LULCC emissions focus on net emissions. However, the typically small net carbon balance represents the small difference between a large carbon uptake and a large carbon loss (Janssens et al., 2005). While the difference between carbon uptakes and losses is more or less averaged out at coarser spatial scales, carbon fluxes in one direction may dominate at the local scale (Fuchs et al., 2016). Therefore, separate tracking of gross carbon emissions and removals can increase the completeness and transparency of LULCC emission assessments (Harris et al., 2021). A commonly used type of emission models is bookkeeping models (e.g., Houghton et al. 1983; Hansis et al. 2015), which use emission factors to calculate the difference in carbon stock following a LULCC (Gasser et al., 2020). They apply specific carbon stock change values for each LULCC type for both vegetation and soil carbon. Usually, bookkeeping models only consider LULCC between natural vegetation, cropland, and pastures, but not between urban or built-up areas. This means that approaches towards spatially explicit estimation of LULCC emissions cannot rely on such bookkeeping models alone. Instead, additional sources are required, such as studies that have estimated the vegetation carbon stock in specific cities (Strohbach & Haase, 2012; Hutyra et al., 2011). Another widespread type of model is dynamic global vegetation models (DGVM) such as the LPJ model (Sitch et al., 2003) which estimate carbon fluxes by modeling processes such as photosynthesis and respiration.

The type and setup of an emission model have a high influence on the estimated emissions, causing great variability among LULCC emission estimates by different models. For Europe, estimates of gross emissions range from 30.4 Tg C yr^-1^ (Smith & Rothwell, 2013) to 41,000 Tg C yr^-1^ (Fuchs et al., 2016) and estimates of net emissions range from -102,000 Tg C yr^-1^ (Houghton & Nassikas, 2017) to -34,000 Tg C yr^-1^ (Gasser et al., 2020). Studies agree that the European net LULCC emissions are currently negative — i.e., a LULCC carbon sink.

All approaches to model LULCC emissions rely on a quantification of LULCC, which is often identified based on remote sensing (Rogan & Chen, 2004; Viana et al., 2019). However, the derivation of LULCC by remote sensing suffers from problems such as the effects of seasonality, which can cause errors for many change detection methods (Verbesselt et al., 2010a). Multiple algorithms have been developed to address this problem. For example, the Breaks for Additive Seasonal and Trend (BFAST) Monitor algorithm (Verbesselt et al., 2010a) and the Continuous Change Detection and Classification (CCDC) algorithm (Zhu & Woodcock, 2014) generate a time series model with seasonality, trend, and break components.

Despite the value of spatially explicit information, estimations of carbon fluxes related to LULCC often only exist at coarse spatial resolution, e.g., one emission value for an entire state, country or even continent (Gasser et al., 2020; Houghton & Nassikas, 2017; Smith & Rothwell, 2013; Gensior et al., 2022). Studies mapping LULCC emissions at a high spatial resolution have been conducted for the tropics (Harris et al., 2012) and globally for forests (Harris et al., 2021). However, they are missing for Europe.

For the spatially explicit estimation of carbon fluxes, events that occur on a small spatial scale such as the construction of individual buildings or cutting of little groups of trees should be recognized. This requires the detection of LULCC at very high spatial resolution. Satellite imagery with sufficient ultra-high spatial resolution such as Maxar or WorldView is available, but it is distributed commercially and therefore costly, prohibiting its use for larger areas outside of specific case studies. The limitations of remote sensing data regarding spatial resolution might be partly overcome using OpenStreetMap (OSM), a global open geo-database that can be used and edited by anyone and is dynamically updated by more than eight million contributors worldwide (OpenStreetMap Contributors, 2022). By providing spatial information at a high level of detail, OSM potentially allows to skip the computation-intensive step of classifying satellite images needed when working with remote sensing data. This can reduce the necessary computation time and power and therefore improve scalability. A comparison of OSM-derived land use and land cover (LULC) information with CORINE landcover has identified a classification accuracy of 76 % (Estima & Painho, 2013). This comparison, however, assumes that the CORINE data set is correct — which can be disputed as OSM mapping might happen at higher spatial resolution and might be more recent. In addition, OSM has a high thematic resolution, facilitating the extraction of a large number of LULC classes which goes beyond the level of detail provided by CORINE. Several approaches have applied OSM for LULC mapping (Yang et al., 2017; Fonte et al., 2017; Schultz et al., 2017), but so far, OSM has not been used to detect or estimate LULCC.

The aim of this study is to estimate the potential of OSM to quantify LULCC-related carbon fluxes to the atmosphere for a regional case study, the German federal state of Baden-Württemberg. To assess the uncertainty by such an approach, the effects of three different levels of pre-processing are compared. In addition, LULCC emissions are estimated using an administrative LULCC product (LaVerDi).

## Materials and methods

### Study area and data

**Fig. 1:**
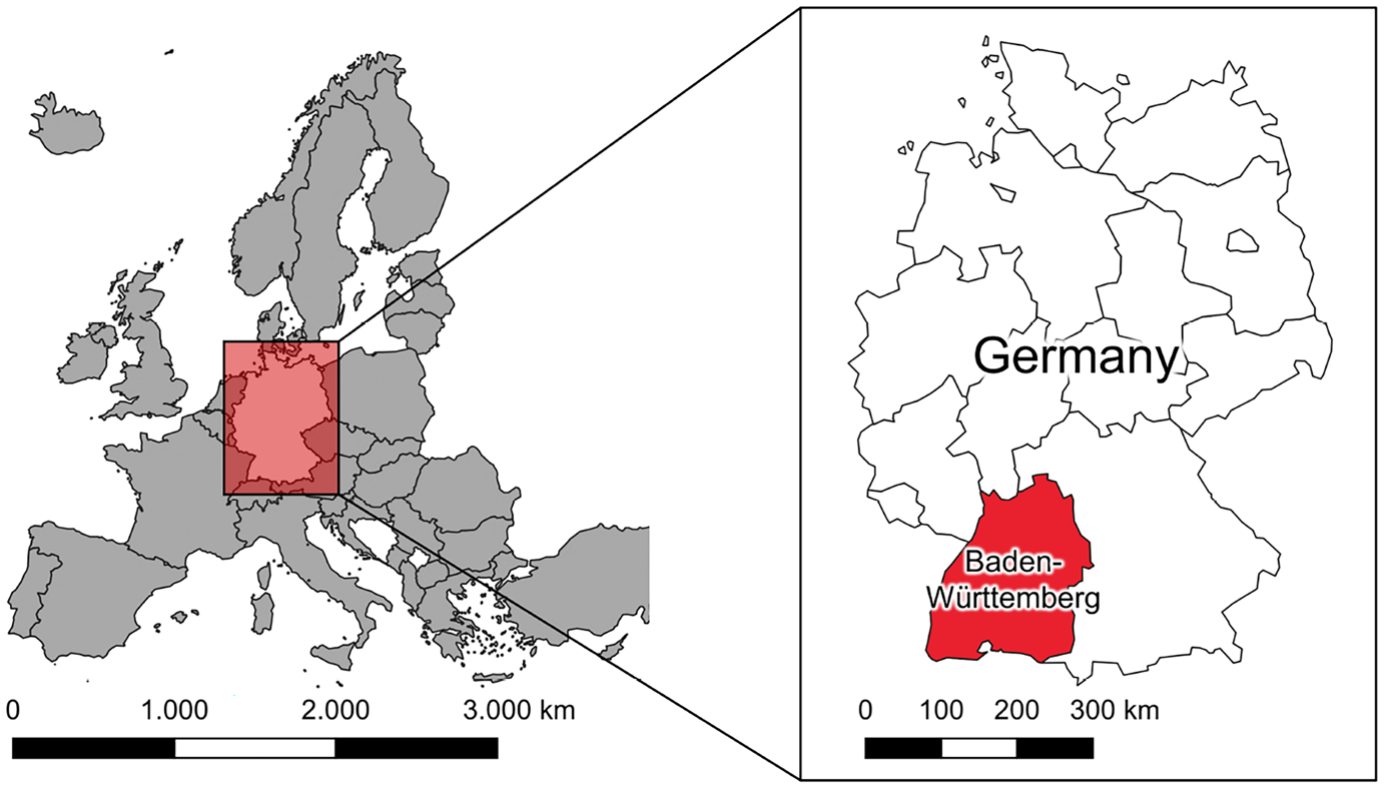
Study area: The federal state Baden-Württemberg located in southwest Germany

The study took place at the German federal state Baden-Württemberg (Fig. 1). The state area is about 35,751 sqkm (State Ministry Baden-Württemberg, 2022), characterized by mosaic landscapes (mixture of forest, meadows, farmland, settlements). With a mean area of 2.54 ha, the size of land use objects is relatively small compared to, e.g., North America. The analysis focuses on the period from March 2018 until October 2019. This period was chosen as one of the LULCC data sources, the administrative dataset LaVerDi, was only available for this period.

**Fig. 2:**
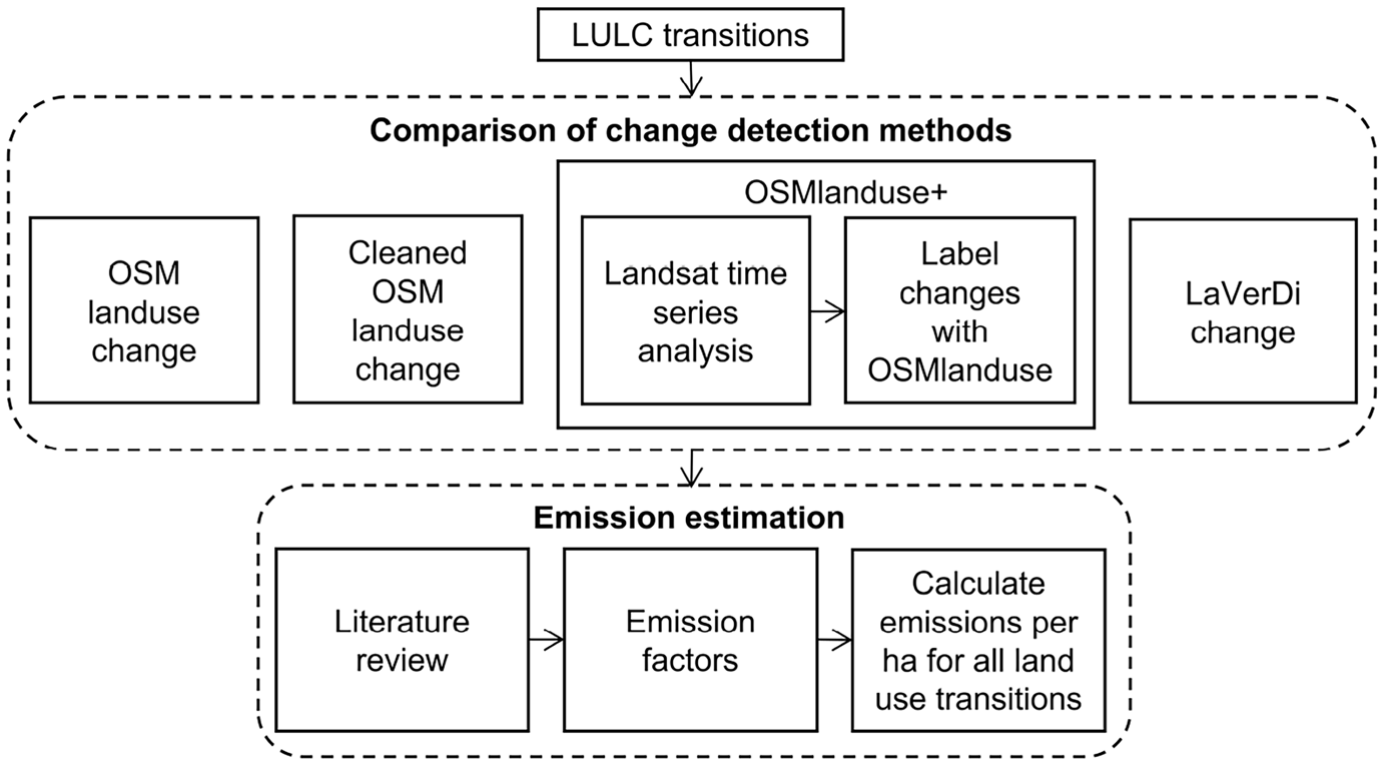
Workflow of the analysis. Change detection and emission estimation were performed spatially explicit, using either OSM polygons or Landsat pixels as the spatial unit

Four different methods to detect LULCC were applied (Fig. 2). Three of the methods (*OSMlanduse*, *cleaned OSMlanduse* and *OSMlanduse+*) used OSMlanduse (Schultz et al., 2017) as the base, a LULC product based on OSM. For OSMlanduse (osmlanduse.org), LULC information from OSM was gap filled by remote sensing derived LULC data (Schultz et al., 2017). For *OSMlanduse+*, OSMlanduse was enhanced by a remote sensing time series analysis. The use of LULCC methods based on OSM is justified, because in Baden-Württemberg, LULC classes obtained from OSM have a high accuracy. Evaluated against an authoritative German reference dataset, the correctness of OSM LULC in the Rhine-Neckar region located in Baden-Württemberg was 95.1 % for forest, 94.8 % for farmland including pastures, and 90 % for urban areas based on data for 2014 (Dorn et al., 2015). Additionally, the LULCC product LaVerDi (Landschaftsveränderungsdienst) from the German Federal Office for Cartography and Geodesy was applied. LaVerDi is based on a Sentinel-2 time series analysis that uses an indicator based on the changes in minimum and mean NDVI for the vegetation period for two reference years. The overall accuracy of the dataset is above 80 % (Bundesamt für Kartographie und Geodäsie, 2022).

### LULCC detection using OSMlanduse

Land use data from OSM were obtained for two points in time: March 2018 and March 2020. The OSM LULC data for 2018 were gathered using the Ohsome API (api.ohsome.org), while the OSM LULC data for 2020 were derived from the OSMlanduse dataset (Schultz et al., 2017), which was compiled based on OSM data of March 2020. The difference between the data acquisition of the second time step of the OSMlanduse data (March 2020) and the remote sensing and the LaVerDi data (October 2019) was considered appropriate for ensuring comparability of the datasets, as OSM requires some time to reflect LULCC, since the change may not be immediately recognized and mapped by the contributors.

After obtaining the OSMlanduse data, the LULC classes used in this work (urban, cropland, pasture, forest) were extracted. In doing so, the OSM key-value pairs of the 2018 OSM data were aggregated to the LULC classes according to the class definitions of the OSMlanduse dataset to match the 2020 OSMlanduse data (Table 1). By comparing the LULC information for the two points in time (2018 and 2020), LULC transitions were derived. The LULC transition areas were then multiplied by the emission factors taken from the literature. Only LULC transitions that lead to carbon fluxes to the atmosphere were considered: cropland to urban, pasture to urban, pasture to cropland, forest to urban, forest to cropland, and forest to pasture.

For *OSMlanduse cleaned*, all change polygons with an area below 0.4 ha (sliver polygons) from the previous step were removed. Such sliver polygons often occur when borders are not well aligned in OSM (Dorn et al., 2015). An auxiliary investigation into the development of the completeness of the LULC information in Baden-Württemberg in OSM during the study period was also done using the ohsome API (Raifer et al., 2019).

**Table 1:** Attribution of OSM key-value pairs to LULC classes in the OSMlanduse dataset

OSM key-value pairs	LULC class
building=garage, landuse=garages, landuse=residential	urban
landuse=farm, landuse=farmland, landuse=farmyard, building=greenhouse, landuse=greenhouse_horticulture	cropland
landuse=meadow	pasture
landuse=forest, landuse=wood	forest

### LULCC detection using OSMlanduse enhanced with satellite images and Landschaftsveränderungsdienst (LaVerDi)

It was assumed that the *OSMlanduse* or *OSMlanduse cleaned* method might overestimate LULCC. The reason is that LULC is not completely mapped in OSM yet. Therefore, LULC objects are still being added to OSM. When a LULC object, e.g., a plot of pasture is added to OSM during the study period in a place where no LULC was mapped before, it will be a change in OSM that most likely does not correspond to a change in the real world. To address these “pseudo-changes”, the uncleaned OSMlanduse changes were refined with a remote sensing time series analysis. LULCC were detected with a Landsat (USGS, 2022) time series analysis from January 2000 until October 2019.

The workflow of the Landsat time series analysis was as follows: First, the Landsat images were downloaded using Google Earth Engine (Gorelick et al., 2017). Clouds and cloud shadows were removed from the images using the cloud bit mask and the cloud shadow bit mask of the Landsat quality assessment band. Next, the images were clipped to the study area of Baden-Württemberg and the spectral indices NDVI, SAVI and NDMI were calculated.

The BFAST Monitor algorithm (Verbesselt et al., 2012) was employed to identify LULCC. It is implemented in R Statistical Software (R Core Team, 2021) and can detect land use changes from time series of satellite images based on a regression model for stable historical behavior. BFAST Monitor requires a historical period of images to generate a regression model describing the long-term trend and the seasonal variation of LULC. The regression model can either be generated for the entire historical period, or only for the stable part of the historical period. When the entire historical data are used, the history period can already include LULCC. This introduces an error into the regression model based on the history and reduces the ability of BFAST Monitor to detect a change during the monitoring period. Using only the stable part of the historical period, on the other hand, ensures that the history period does not contain any LULCC (Verbesselt et al., 2012).

The stable part of the historical period can be derived using a reverse-ordered cumulative sum test (ROC). Starting backwards from the beginning of the monitoring period, the ROC method sums up the spectral index values for each pixel. The history period is considered stable as long as the ROC increases steadily. As soon as the ROC stops to rise steadily, a break is detected. This point in time is then considered to mark the beginning of the stable history period.

BFAST Monitor fits a regression model to the historical period for each pixel in the given time series of images. The regression model was specified by a linear trend and a harmonic seasonal component. The harmonic seasonal component uses up to three harmonic terms, which leads to a more robust detection of phenological changes during the time series as opposed to a linear seasonal model (Verbesselt et al., 2010b). After fitting the regression model, BFAST Monitor detects LULCC by comparing the observed data in the monitoring period to the regression model (Verbesselt et al., 2010a). A LULCC event is attributed to all pixels where the difference between the model and the observed data exceeds a certain threshold.

For the BFAST Monitor analysis, the following spectral indices were applied: Normalized difference vegetation index (NDVI) for the class pasture, normalized difference moisture index (NDMI) for forest, and soil-adjusted vegetation index (SAVI) for cropland. Based on the hypothesis that each index is related to a LULC class, each index was used exclusively for a specific LULC type. Because it is also a LULCC dataset based on remote sensing data, LaVerDi was used as a measure to find the best BFAST Monitor settings. Despite also exhibiting uncertainties, the LaVerDi dataset was deemed suitable for this task. For each initial LULC class, a test area was chosen based on the occurrence of all relevant LULC transitions with that initial LULC class in the LaVerDi change data. The test areas were at different locations in Baden-Württemberg. For each initial LULC class, the settings where the BFAST Monitor changes had the highest overlap and minimal mismatch with the LaVerDi changes were derived.

The period from January 2000 until February 2018 constituted the historical period and the period from March 2018 until October 2019 constituted the monitoring period. During testing, the following BFAST Monitor parameters were varied: The order of the harmonic term of the regression model (order) and the method to determine the stable part of the historical period (history). The order was varied between 1, 2, and 3. For the history, either an ROC was applied or the entire historical period was used (all).

As result of the tests, the following best-suited BFAST Monitor settings were derived: order of 3 and history method ROC for the initial class cropland, and order of 2 and history method ROC for the initial classes pasture and forest. These settings were applied for the BFAST Monitor analysis in the entire state of Baden-Württemberg. Subsequently, the resulting BFAST Monitor change areas were intersected with the areas of the uncleaned OSMlanduse changes from each initial LULC class to all other emission relevant classes. All areas contained in BFAST monitor changes and OSMlanduse changes then constituted the OSMlanduse+ changes.

As fourth change detection method, the LaVerDi LULCC product (March 2018 until October 2019) was used as provided by the Laverdi website (Bundesamt für Kartographie und Geodäsie, 2022). However, all polygons that were flagged as “not relevant” by the confidence attribute of the dataset were removed. Subsequently, the change polygons of the emission relevant LULC transitions were extracted from the LaVerDi change areas.

### Estimation of carbon fluxes

LULCC-induced CO_2_ emissions were estimated as “committed fluxes”. This assumes that the carbon stocks before and after a LULCC are in an equilibrium and does not account for the temporal dimension of delayed changes (Hansis et al., 2015). Three different methods were used to estimate the emissions. For each type of LULC transition, e.g., forest to cropland, forest to urban, etc. a specific emission factor was used to derive the carbon fluxes in Mg C ha^-1^ (Table 2). The emission factors were derived by assigning a prescribed carbon stock to each LULC class and calculating the difference between the carbon stocks for each LULC transition.

**Table 2:** Emission factors used for the different carbon flux attribution methods. Methods 1 and 3 relied on the emission factors from the BLUE model and method 2 augmented BLUE emission factors by information from the Carbon Dioxide Information Analysis Center. Method 3 mainly differs from the other methods in that it assumed that there is no soil carbon in urban areas, while methods 1 and 2 used a soil carbon estimation from a case study

LULC transition	Carbon fluxes [Mg C ha^-1^]		
	Method 1	Method 2	Method 3
Cropland to urban	1.0	35.0	61.0
Pasture to urban	36.5	36.5	96.5
Pasture to cropland	35.5	1.5	35.5
Forest to urban	149.0	156.0	198.0
Forest to cropland	148.0	155.0	197.0
Forest to pasture	112.5	119.5	161.5

The carbon stocks were primarily derived from the BLUE model (Hansis et al., 2015), which assigns different emission factors to different types of LULC transition (methods 1 and 3). The BLUE model considers the removal or accumulation of carbon in the vegetation as well as changes in soil carbon. Carbon stocks from a database of the Carbon Dioxide Information Analysis Center (Houghton & Hackler, 2001) were applied additionally (method 2). To estimate the carbon stock in urban areas, a vegetation carbon estimation of Leipzig (Strohbach & Haase, 2012) combined with a soil carbon estimation for suburban areas in England (Bradley et al., 2005) was used. However, the soil carbon estimation was only used in methods 1 and 2, while in method 3 the assumption was made that there is no soil carbon in urban areas. The study by Strohbach and Haase (2012) was chosen because the study site Leipzig is a city in Germany, whose vegetation density was assumed to be similar to that of urban areas in Baden-Württemberg.

## Results

### Strong variations of carbon flux estimates were mostly caused by the LULCC detection method

**Fig. 3:**
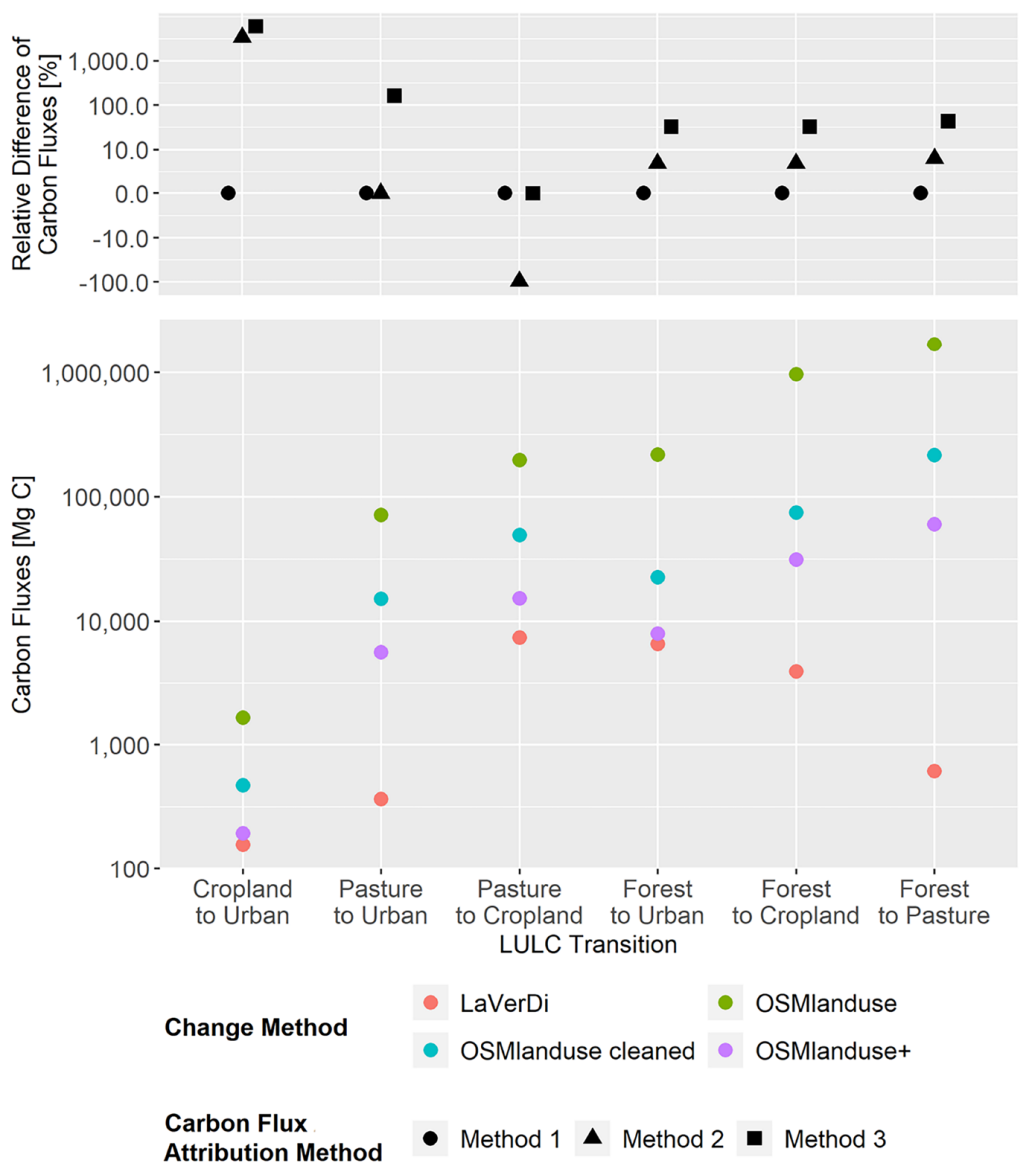
Total carbon fluxes related to LULCC in Baden-Württemberg between March 2018 and October 2019 (LaVerDi, OSMlanduse+), and March 2018 and March 2020 (OSMlanduse, OSMlanduse cleaned). The upper plot shows the relative difference of carbon fluxes [%] with respect to LULC transition and carbon flux attribution method. The carbon fluxes of method 1 are set to 0. The lower plot shows the total absolute carbon fluxes [Mg C] calculated with carbon flux attribution method 1 with respect to LULC transition and LULCC method

The estimated carbon fluxes by LULCC for the whole of Baden-Württemberg varied by several orders of magnitude depending on the method used to detect LULCC and the carbon flux attribution method (Fig. 3). The total LULCC emissions ranged from 3,100,000 Mg C to 4,500,000 Mg C for the OSMlanduse change method, from 370,000 Mg C to 560,000 Mg C for the cleaned OSMlanduse change method, from 120,000 Mg C to 180,000 Mg C for the OSMlanduse+ change method, and from 18,000 Mg C to 33,000 Mg C for the LaVerDi change method. However, the effect of the change method on the carbon fluxes was much higher than the effect of the carbon flux attribution method. In other words, the differences in the derived LULCC areas had a stronger effect than the applied emission factors.

The differences of LULCC estimated by the different methods had an especially strong effect on the carbon fluxes for the LULCC from forest to all other classes (Fig. 3). At the same time, the effect of the carbon flux attribution method on the carbon fluxes was mostly relevant for LULCC with the initial classes cropland and pasture. The carbon flux attribution method led to a variation of the estimated fluxes by 61 times for the LULC transition cropland to urban, by 2.6 times for the LULC transition pasture to urban and by 24 times for the LULC transition pasture to cropland. Conversely, the effect of the carbon flux attribution method on the fluxes for the LULC transitions with the initial class forest was rather low. The carbon flux attribution method led to a variation of the estimated fluxes by 1.3 times for the LULC transitions forest to urban and forest to cropland and by 1.4 times for the LULC transition forest to pasture.

### OSMlanduse changes were strongly influenced by sliver polygons

**Fig. 4:**
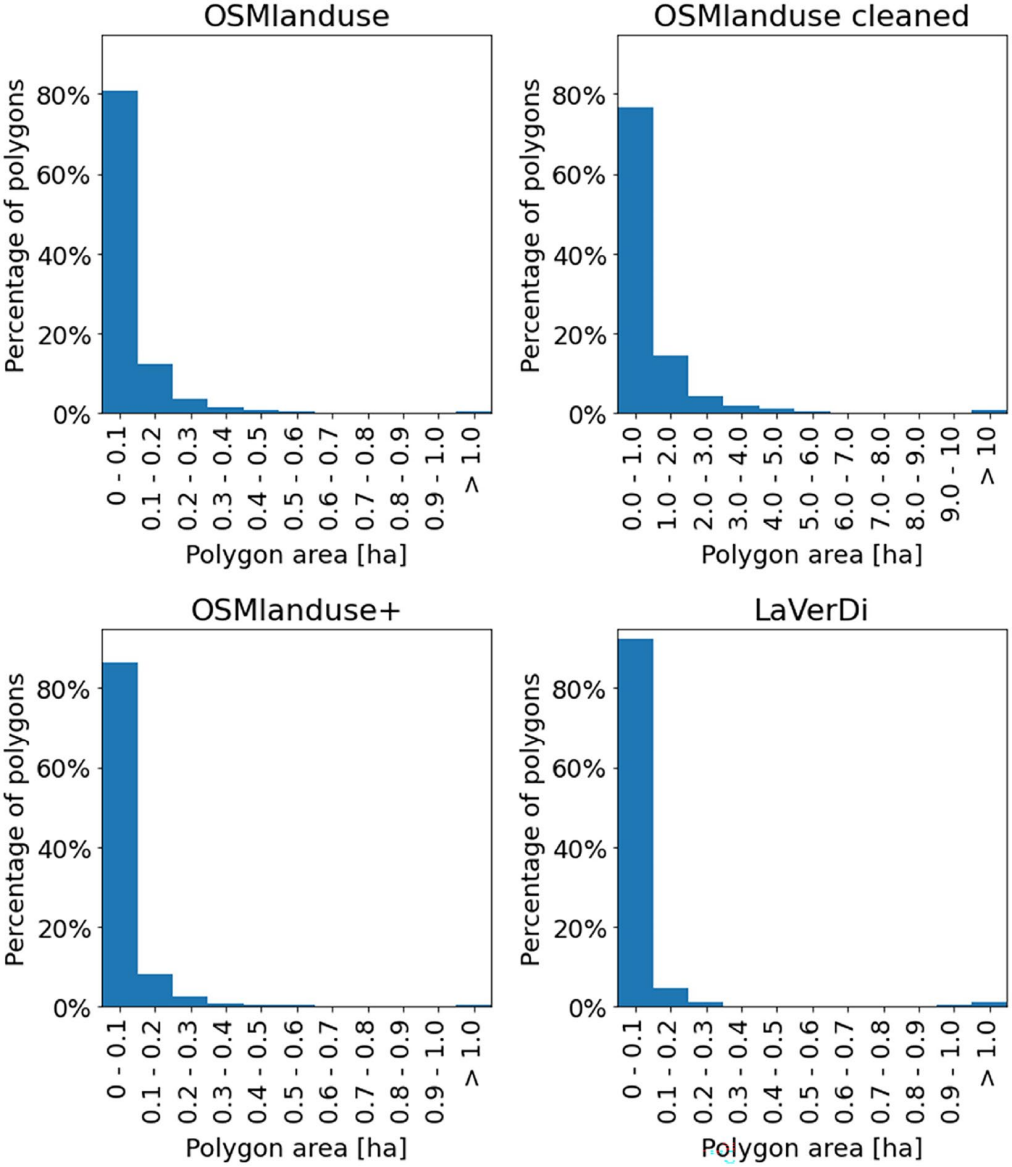
Histograms of polygon areas [ha] of the LULCC methods

The mean change polygon areas of all LULCC methods were similar — always below 0.2 ha. However, all LULCC methods showed a similar skewed distribution of polygon sizes with a high number of very small polygons and only a limited number of larger polygons over 0.4 ha in size (Fig. 4). Moreover, the share of small polygons with a size up to 0.4 ha of the entire change area was different for each LULCC method. While polygons with an area up to 0.4 ha only accounted for a small area in the LaVerDi changes, they made up a higher percentage of the OSMlanduse+ change area and even accounted for the majority of the entire OSMlanduse change area (Table 3).

These small polygons with an area up to 0.4 ha can presumably be considered as sliver polygons (Dorn et al., 2015; Mas, 2005) in most cases, causing the detection of much pseudo change with the OSMlanduse change method. Depending on the LULC transition, the cleaned OSMlanduse carbon fluxes were between 7.8 % and 28.6 % of the OSMlanduse carbon fluxes. This shows that sliver polygons accounted for the majority of OSMlanduse change carbon fluxes across all LULC transitions.

**Table 3:** LULCC area [ha] and area share of change polygons $\le$ 0.4 ha [%] for the different LULCC detection methods

LULCC detection method	Change area [ha]	Area share of change polygons $\le$ 0.4 ha [%]
OSMlanduse	32,160	71.3
OSMlanduse cleaned	4,867	0
OSMlanduse+	1,577	36.2
LaVerDi	453	6.8

### LULCC areas differed strongly depending on LULCC detection method

For all LULC transitions in the federal state of Baden-Württemberg, the OSMlanduse change areas were largest, the LaVerDi change areas were smallest, and the OSMlanduse+ change areas were in between (Table 3). In line with that, the total areas for cleaned OSMlanduse change were between the OSMlanduse and the OSMlanduse+ change areas (Table 3). For the LULC transitions with the initial class forest, the differences of the LULCC areas between the different change methods were higher than for the other LULC transitions.

The proportions of the change areas of the different LULC transitions also varied according to the change method. For the LULCC products derived from OSMlanduse, the LULC transition forest to urban had the smallest change area and the LULC transition forest to pasture had the largest change area. For the LaVerDi change method, the LULC transition forest to pasture had the smallest change area and the LULC transition pasture to cropland had the largest change area.

### Spatial patterns of LULCC

**Fig. 5:**
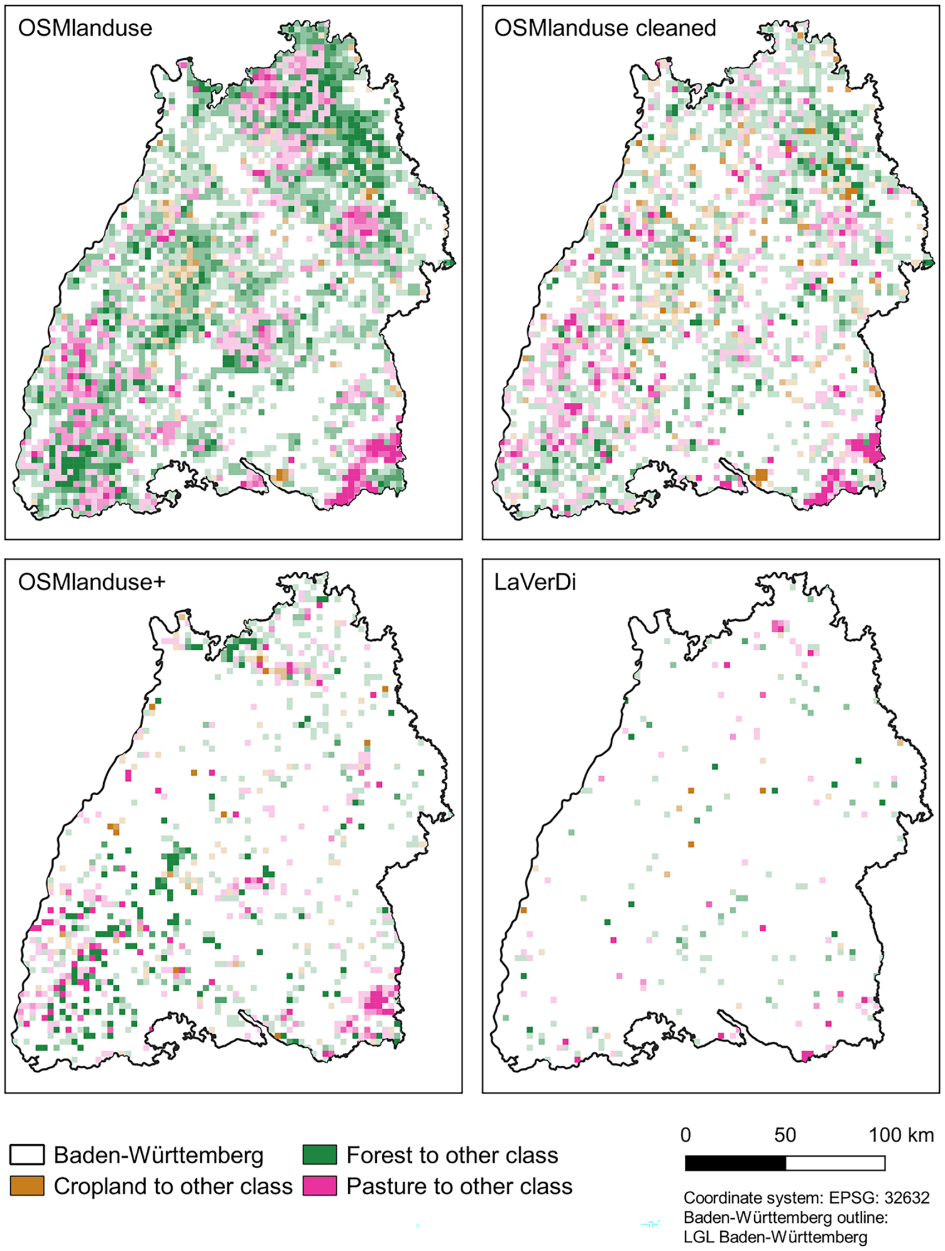
LULCC in Baden-Württemberg derived with the four different LULCC methods per 3 x 3 km grid cell. Each grid cell is colored by the dominant LULCC type. Grid cells with a larger LULCC area are indicated by a darker (less transparent) color value

Of all LULCC methods, the OSMlanduse approach led to the strongest LULCC (Fig. 5). Most of the OSMlanduse changes had the initial classes pasture and forest, while changes with the initial class cropland were less prevalent. The OSMlanduse change areas were not distributed evenly throughout the federal state. Rather, there were some regions without any change and there were regional clusters where certain LULC transitions were dominant, e.g., forest to other classes in the northeast and pasture to other classes in the southeast. The regional clusters mentioned above for the OSMlanduse changes were still partially visible in the cleaned OSMlanduse changes.

The change polygons detected with the OSMlanduse+ change method were more dispersed than with the cleaned OSMlanduse change method. Especially for the transitions from forest to other classes, almost all detected changes were isolated pixels. For the initial class cropland, the detected changes were scarce. Albeit less pronounced than in the OSMlanduse and the cleaned OSMlanduse changes, the cluster of changes from pasture to other classes in the southeast of Baden-Württemberg was still visible (Fig. 5). The change areas detected with the LaVerDi change method were mostly unconnected and did not show any regional clusters (Fig. 5).

## Discussion

### Comparison of carbon flux estimates to other studies

The carbon flux estimates of the LULCC methods OSMlanduse cleaned, OSMlanduse+, and LaVerDi are in line with the results of other studies, while the OSMlanduse change method seems to overestimate the emissions (Table 4). Here, it is important to note that with one exception, all studies available for comparison have been conducted at coarser spatial scale — i.e., at the national or (sub-) continental scale (c.f. Table 4). For those studies where LULCC detection was conducted at a coarser spatial scale, the results are only partially comparable.

**Table 4:** Comparison of mean annual LULCC carbon flux estimates to estimates from the literature: Statistisches Landesamt Baden-Württemberg (2022) (1), Gensior et al. (2022) (2), Fuchs et al. (2016) (3), Gasser et al. (2020) (4), Houghton & Nassikas (2017) (5), Smith and Rothwell (2014) (6). Absolute emission estimates were converted to carbon fluxes per hectare to enable comparison. The change methods of this study only cover 77.8 % of Baden-Württemberg. This is partly due to the incompleteness of mapped LULC in OSM and partly because this study only considered LULCC between the classes cropland, forest, pasture, and urban. The change methods of this study covered 93 % of the LULC mapped in OSM

Reference area	Emissions category	Urban included	Source	Reference area [km^2^]	C fluxes [Mg C yr^-1^]	C fluxes [Mg C ha^-1^ yr^-1^]	Reference period
77.8 % of Baden-Württemberg	gross	yes	OSMlanduse	27,823	2,412,551^a^	0.867	03/2018–03/2020
77.8 % of Baden-Württemberg	gross	yes	OSMlanduse cleaned	27,823	291,710^a^	0.105	03/2018–03/2020
77.8 % of Baden-Württemberg	gross	yes	OSMlanduse+	27,823	93,591^a^	0.034	03/2018–09/2019
77.8 % of Baden-Württemberg	gross	yes	LaVerDi	27,823	15,599^a^	0.006	03/2018–09/2019
Baden-Württemberg	net	yes	1	35,751	-1,925,910^d^	$-$0.539	2018–2019
Germany	gross	yes	2	357,022	11,853,000^d^	0.332	2018–2019
Europe^b^	gross	no	3	4,452,789	41,000,000	0.092	1950–2010
Europe^c^	net	partly	4	5,800,000	-34,000,000	$-$0.059	2009–2018
Europe^c^	net	no	5	5,800,000	-102,000,000	$-$0.176	2006–2015
Western Europe	gross	yes	6	4,677,475	30,411	6.5 $\times$ 10^-5^	2020

^a^Mean fluxes of the three different carbon flux attribution methods for each LULCC method

^b^European Union without Croatia plus Switzerland and the UK

^c^Without Greenland and states of the former Soviet Union. For a list of countries included, see Houghton & Nassikas (2017), Table 2

^d^Emissions were given in CO_2eq_ yr^-1^ and converted to C yr^-1^ in the following way: C yr^-1^ = CO_2eq_ yr^-1^ $\cdot$ 0.27

The comparability of this study to other studies is also limited by different methodologies. Many other studies estimate net emissions, while this study assessed gross emissions. While this limits a direct comparison of the emissions, their magnitudes can still be discussed and compared. Table 4 shows three studies assessing net emissions from LULCC in Europe (Statistisches Landesamt Baden-Württemberg, 2022; Gasser et al., 2020; Houghton & Nassikas, 2017). Aside from estimating net emissions instead of gross emissions, these studies used a comparable approach as this study. They also used a bookkeeping approach incorporating carbon stock values for vegetation and soil carbon and they also modeled carbon stock changes at discrete time steps. All three studies deduced a LULCC carbon sink, shown by the negative carbon fluxes — implying that the LULCC emissions were more than compensated by carbon sequestration in forests. However, the range between the estimates of the studies is very high (Table 4).

The high range of net carbon emission estimates is probably caused by methodological differences between the studies. For example, Statistisches Landesamt Baden-Württemberg (2022) does not only estimate LULCC emissions, but also the emissions occurring permanently in stable LULC classes. These include carbon fluxes from organic soils to the atmosphere in the LULC classes pasture, cropland, and wetlands. Conversely, Gasser et al. (2020) and Houghton & Nassikas (2017) only consider carbon fluxes caused by LULCC, as is done in this study. The high amount of biomass in the forests which permanently sequesters a lot of carbon could be a reason why the estimated carbon sink in Statistisches Landesamt Baden-Württemberg (2022) was more pronounced than in Gasser et al. (2020) and Houghton & Nassikas (2017).

The estimates of gross carbon emissions caused by LULCC for Germany and Europe range from 6.5 $\times$ 10^-5^ Mg C ha^-1^yr^-1^ to 0.332 Mg C ha^-1^yr^-1^ (Table 4). These studies have in common that, as in this work, they derive spatially explicit LULCC. The carbon flux estimates of the LULCC methods OSMlanduse cleaned, OSMlanduse+, and LaVerDi were within the range of the other studies assessing gross emissions, while the OSMlanduse change method seems to have overestimated the emissions.

Similarly to the net emission studies, the carbon flux estimates of the gross emission studies also have a high range (Table 4). Several methodological differences between the studies, which may influence the carbon flux estimates, can be identified, e.g., the type of model used. As in this study, Fuchs et al. (2016) and Gensior et al. (2022) used a bookkeeping approach incorporating carbon stock values for vegetation and soil carbon. Conversely, Smith and Rothwell (2013) used a dynamic vegetation model, which could be a reason for their much lower carbon flux estimate. The carbon fluxes estimated with the cleaned OSMlanduse change method and the OSMlanduse+ change method were in line with the estimates of the studies also using a bookkeeping approach (Fuchs et al., 2016; Gensior et al., 2022), while the fluxes estimated with the LaVerDi change method were much smaller (Table 4).

Another methodological difference between the studies is the question whether or not the temporal dimension of carbon stock changes is included. While this study and the study by Fuchs et al. (2016) include emissions in the longer term, assuming that the carbon stocks change immediately after a LULCC, Smith and Rothwell (2013) separate their carbon stock changes into short-term and long-term carbon stock changes. This means, e.g., that when a forest is harvested, not all vegetation carbon is transferred to the atmosphere directly. Instead, some of it, like slash and dead roots, may stay in its original pools for a longer period of time and some of it may be transferred to other pools such as wood product pools. The estimated carbon fluxes of Fuchs et al. (2016) and this study were much higher than the carbon flux estimated by Smith and Rothwell (2013) (Table 4). Using a “committed fluxes” approach presumably leads to higher gross emission estimates, because it does not account for the temporal lag of carbon fluxes following a LULCC, thus increasing the estimated carbon fluxes to the atmosphere resulting directly from a LULCC.

Finally, the question whether or not to include urban areas may influence the carbon flux estimates. At present, LULC transitions from other classes to urban are much more common in Europe than LULC transitions from urban to other classes. LULC transitions from other classes to urban usually lead to carbon fluxes to the atmosphere. Therefore, including urban areas presumably leads to higher carbon flux estimates than excluding them. Hence, a reason why the carbon flux estimate by Gensior et al. (2022) is higher than the estimate by Fuchs et al. (2016) (Table 4) could be that like this study, Gensior et al. (2022) include urban areas while Fuchs et al. (2016) do not.

### OSMlanduse overestimates carbon fluxes due to mapping activity during study period and sliver polygons

Gasser et al. (2020) showed that carbon flux estimates between two studies can differ significantly, even if both studies used the same carbon densities. This suggests that ways of processing and implementing LULCC data sets have significant influence on the derived carbon fluxes (Gasser et al., 2020; Smith & Rothwell, 2013). The strong effect of the change detection method on the carbon flux estimates in this study confirmed this suggestion. This important effect was mainly caused by the difference between the sizes of the change areas identified by the different LULCC methods. The OSMlanduse changes included many changes representing the addition of objects, change of OSM tags or adaptation of shapes that did not represent actual LULCC. This explains the observation made in the previous section that with the OSMlanduse change method, LULCC were probably overestimated as compared to the gross emissions estimated in the other studies (Fuchs et al., 2016; Smith & Rothwell, 2013; Gensior et al., 2022) (Table 4). One assumes that features keep being added to OSM until the most of the mapping, with respect to a selected feature type, has been completed (Rehrl & Gröchenig, 2016). Our auxiliary investigation of the LULC mapping development in Baden-Württemberg using the ohsome API (Raifer et al., 2019) showed that especially in rural areas, there were still considerable gaps in the LULC coverage, but they were gradually being filled, confirming this assumption.

The share of the sliver polygons of the total change area in the OSMlanduse+ changes was probably smaller than in the OSMlanduse changes, since many of the sliver polygons were removed because no change was detected there by BFAST Monitor. Removing all change polygons with an area below 0.4 ha from the OSMlanduse changes reduced the estimated carbon fluxes to a level where they are comparable to other studies such as Fuchs et al. (2016) (Table 4). This indicates that to dispose of the false positive LULCC from OSMlanduse, a computation- and data-intensive remote sensing time series analysis may not be strictly necessary. However, a polygon area threshold to remove sliver polygons may fail to remove some of the sliver polygons and probably also delete some polygons that are not sliver polygons. It could be fruitful to test more sophisticated methods of removing sliver polygons than the simple polygon area threshold, e.g., by using the mean width of the polygons as a metric to identify slivers (Mas, 2005) or by means of a supervised classification (Delafontaine et al., 2009).

### Effect of change method on fluxes is strongest for initial LULC class forest

The observed effect of the change method on the estimated carbon fluxes was strongest for changes from forest to all other classes. This was partly caused by especially high differences in change area between the different change methods for LULC transitions with the initial class forest. It was also caused by a relatively low effect of the carbon flux attribution method, as carbon fluxes were always high when forest was transformed to a different LULC class. This is not unexpected, since forest stores much more carbon compared to the other LULC classes (Hansis et al., 2015).

### Choice of carbon stock values influences carbon flux estimates

Carbon stock estimates per LULC class have been identified as a major source of uncertainty for the estimation of LULCC triggered carbon fluxes (Gasser et al., 2020; Houghton & Nassikas, 2017). This is in line with our results. However, our findings indicate that the importance of this source of uncertainty differed between the different LULC transitions. The effect of the carbon flux attribution method on the flux estimates was low for LULC transitions with the initial class forest. A possible explanation is that the conversion of forest to other LULC classes always leads to high carbon fluxes, no matter which carbon flux attribution method is used. This is in line with findings from a study at small scale (Fuchs et al., 2016).

This important effect of the carbon flux attribution method for the LULC transitions pasture to cropland, pasture to urban, and cropland to urban can be presumably partly attributed to different estimations regarding the amount of carbon stored in soil under cropland (Houghton et al., 2001; Hansis et al., 2015). The estimations differ, because the amount of carbon stored in cropland soil depends on the climate and on the land management. This includes, but is not limited to, fertilization, type of crop, crop rotation, and tillage practices (Lal, 2002). Depending on how much carbon is stored in cropland soil, the estimated carbon fluxes for a conversion from pasture to cropland can differ greatly. This uncertainty in carbon loss and gain from pasture is also mentioned in Smith and Rothwell (2013).

Another uncertainty is linked to the different assumptions regarding the carbon stock in urban soil. If it is assumed that surfaces in urban areas are completely impervious, there is much less soil carbon (Raciti et al., 2012), and the urban carbon stock is much lower than the carbon stock of cropland. In that case, a LULCC generates a high carbon flux. If it is assumed on the other hand, that urban areas also include many non-impervious surfaces such as parks or private gardens, there is a considerable soil carbon stock as well (Bradley et al., 2005). In this case, the carbon flux of a LULCC from cropland to urban is close to zero. In Gensior et al. (2022), urban areas were assumed to consist to 50 % of non-impervious surfaces. For these surfaces, soil carbon stocks of grassland were used (Gensior et al., 2022), which is in line with carbon flux attribution methods 1 and 2 of this study.

It is also important to acknowledge that the carbon stocks assigned to the different LULC classes are mean values and cannot reflect the spatial heterogeneity of the LULC classes in an entire region (Wiedinmyer et al., 2011). While this is less problematic for emission estimates over large areas, where over- and underestimations of local LULCC emissions tend to average out, it can cause considerable inaccuracies for the estimated emissions of an individual LULCC change. This is shown by an example of forest fires in Siberia, where the amount of carbon stored in the local ecosystem and the severity of the specific fire event can lead to a 50 % variation of direct carbon emissions (Soja et al., 2004). It can be assumed that this variability of LULCC emissions within a LULC class is higher, the broader the class. This means that in order to obtain more reliable estimates of local, spatially explicit LULCC emissions, future studies should try to use more fine-grained LULC classes with emission factors that are as representative for the local ecosystem as possible. Perhaps the carbon stocks assigned to the LULC classes could also be improved by applying a DGVM such as the LPJ model (Sitch et al., 2003) to simulate the carbon stocks in Baden-Württemberg.

As discussed in the “Comparison of carbon flux estimates to other studies” section, the estimation of carbon fluxes as “committed fluxes”, as it was done in this study, does not account for the temporal lag of carbon fluxes related to LULCC (Hansis et al., 2015). In future studies, emission attribution methods that also consider the delay of carbon fluxes caused by LULCC could be used.

### Relation of OSMlanduse change and completeness

It has been shown that the completeness of OSM is influenced by the degree of urbanization. In general, OSM mapping is more complete in densely populated urban areas than in rural areas (Arsanjani et al., 2015; Zia et al., 2019; Zielstra & Zipf, 2010) and these regional differences also apply in Baden-Württemberg (Brückner, 2020). Our auxiliary investigation of the development of LULC completeness showed that in many rural areas, the completeness of OSM LULC during our study period differed by LULC class. While the classes urban and forest already had a high completeness in 2018, the classes cropland and pasture were still quite incomplete, but exhibited considerable mapping activity during our study period. In areas with lower LULC completeness, there seemed to be more OSMlanduse changes than in areas with higher LULC completeness. Analyzing whether there is a generalizable correlation between OSMlanduse completeness and the magnitude of OSMlanduse changes is a potential topic for a future study. Presumably, the occurrence of certain LULC transitions also depends on the occurrence of the respective LULC classes, as some classes are more completely mapped than others.

### Discussion of the LaVerDi change dataset

As LULCC detection from satellite images is a complex task exhibiting various uncertainties, the LaVerDi change dataset inevitably is also subject to errors. Although LaVerDi was — despite its errors — deemed suitable as a measure to find the best BFAST Monitor settings, its limitations are addressed shortly in this section. One error source may be the complex spectral pattern of the class cropland, which makes it difficult to reliably distinguish this class from other LULC classes with remote sensing (Viana et al., 2019). Many of the LaVerDi changes were changes from the classes cropland to “sand/rock/soil” or “open spaces with/without vegetation”. These changes probably represent fields that were green in one year and bare in another year, meaning the LULC class remained cropland. The reason why LaVerDi classified these cases as LULCC may lie in its methodology: LaVerDi compares metrics describing greenness such as NDVI between the same period of two reference years (Bundesamt für Kartographie und Geodäsie, 2022). If a field is planted and therefore green in year one and bare in year two, LaVerDi will detect a change. BFAST Monitor on the other hand, with its consideration of the historical period, will learn that these changes between green and bare ground often occur on cropland and will not identify a change. BFAST Monitor only detects a change if there is a deviation from the usual pattern of planting, growing, ripening, and harvest, such as a conversion to built-up area.

Another aspect to consider is that the spectral signatures of pasture and cultivated cropland can be similar (Rodrigues et al., 2020). Therefore, changes from pasture to cropland may have been missed by both LaVerDi and BFAST Monitor. Considering the limitations of LaVerDi, it may be feasible to map LULCC in the test areas manually with satellite images and use that as guidance to derive the best-suited BFAST Monitor settings in a future study instead of using LaVerDi as guidance.

## Conclusion

The study showed that OSM can be used to detect LULCC and to estimate LULCC emissions. However, the suitability of the different change methods varied strongly. It is important to remove sliver polygons from the detected LULCC, as they were the main factor causing the overestimation of change with the OSMlanduse change method. Many of the pseudo change areas could also be removed from the OSMlanduse changes by refining the change detection with BFAST Monitor using a satellite image time series. However, it is advisable to use the cleaned OSMlanduse change method because while the OSMlanduse+ method added a lot of methodological complexity and required processing time to the analysis, it did not have any significant advantages over the cleaned OSMlanduse method regarding its ability to remove sliver polygons. For the initial LULC classes cropland and pasture, the carbon flux estimates were strongly influenced by the selected carbon stock values, showing that the choice of carbon stock values for these classes should be treated with care. Since the carbon stocks of cropland and pasture depend on the local climate and management practices, these carbon stock values should be adapted to the study area if possible.

The approach of this study can be used to calculate the absolute carbon fluxes related to LULCC for a given area or any specific LULCC. It also provides explicit spatial information about carbon fluxes related to LULCC. In doing so, it can help to increase the knowledge about local drivers of climate change, which is a prerequisite for measures to reduce LULCC emissions and mitigation strategies on the local scale.

In future studies, it could be fruitful to test more sophisticated methods of removing sliver polygons than the simple polygon area threshold. Another topic for a future study could be to analyze whether there is a generalizable correlation between OSMlanduse completeness and the magnitude of OSMlanduse changes. The emission attribution methods could be adapted by making more realistic assumptions, e.g., also considering the delay of carbon fluxes caused by LULCC. Uncertainties caused by the spatial heterogeneity of the LULC classes could perhaps be reduced by applying a DGVM such as the LPJ model (Sitch et al., 2003) to simulate the carbon stocks in Baden-Württemberg.

## Data Availability

The datasets generated during and/or analyzed during the current study are available from the corresponding author on reasonable request.

## Code availability

The code used in the current study is available here: https://github.com/GIScience/OSMlanduse-BFM.
